# Giant retroperitoneal liposarcoma during pregnancy: a case report

**DOI:** 10.1186/s12957-015-0555-0

**Published:** 2015-04-10

**Authors:** DongFang Huo, Lin Liu, Yun Tang

**Affiliations:** Department of General Surgery, Beijing Tsinghua Changgung Hospital, Medical Center, Tsinghua University, NO. 168 Litang Road, Changping District Beijing, 102218 China; Department of General Surgery, PLA General Hospital, No. 28, FuXing Road, Beijing, 100853 China

**Keywords:** Retroperitoneal tumor, Myxoid liposarcoma, Pregnancy

## Abstract

**Background:**

The peak prevalence of retroperitoneal liposarcoma (RPLS) is between the age of 40 to 70 years, usually seen in male 1.43:1. RPLS during pregnancy is extremely rare and a challenge for both the surgeon and the pregnant woman.

**Case presentation:**

A 27-year-old woman was discovered to have a giant retroperitoneal tumor incidentally during her routine obstetric examination at 16 weeks of gestation. The en bloc resection was performed with preservation of the fetus at 20 weeks of gestation and the final pathology was consistent with low-grade myxoid liposarcoma. The patient had an uneventful pregnancy course with a termed delivery. A CT scan was taken 6 months following the surgery, and no local recurrence was detected.

**Conclusion:**

With a thorough consultation and multidiscipline collaboration, en bloc resection of retroperitoneal liposarcoma with preservation of the fetus could be feasible in the late second trimester.

## Background

The peak prevalence of retroperitoneal liposarcoma (RPLS) is between the age of 40 to 70 years, which is usually seen in male 1.43:1 [1,2]. RPLS during pregnancy is extremely rare and a challenge for both the surgeon and the pregnant woman [3]. We are presenting such a case; en bloc resection of RPLS was performed at 20 weeks of gestation with the preservation of the fetus.

## Case presentation

**Figure 1:**
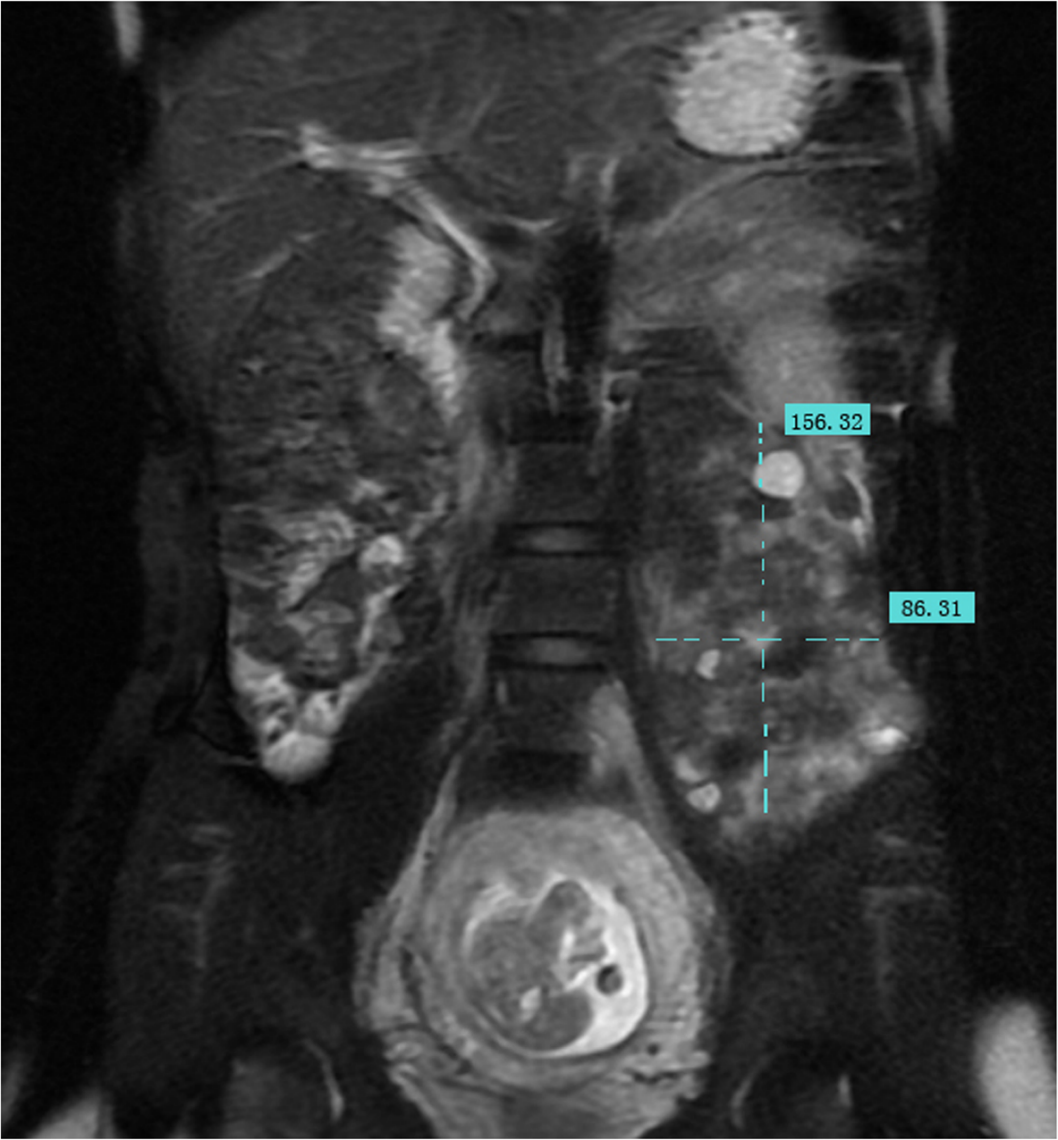
**MRI (Coronal T2-FRFSE Fat SAT).** The extend of the tumor and relation with adjacent organs (1).

**Figure 2:**
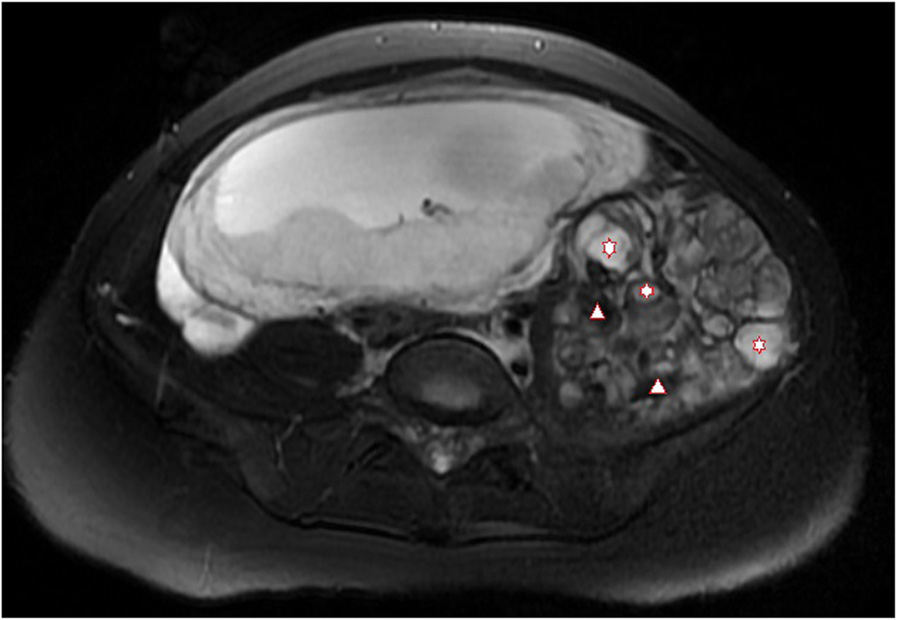
**MRI (Axial T2-FRFSE Fat SAT).** The well-circumscribed multinodular mass with heterogeneous intensity of this retroperitoneal liposarcoma. Star for tumor, triangle for gelatinous components (2).

A 27-year-old woman was discovered to have a giant retroperitoneal tumor incidentally during her routine obstetric examination at 16 weeks of gestation. Ultrasonographies demonstrated a giant left retroperitoneal tumor measuring 21 × 8 cm that extended from the lower border of the left kidney to the left pelvis. An abdominal routine MRI revealed the giant left retroperitoneal tumor, measuring 16 × 9 cm that extended from the hilum of the left kidney to the left pelvis, the lesion had a close relation with the psoas and iliopsoas, and the left kidney and uterus were compressed laterally. There was no liver metastasis and urinary system and major vessel involvement (Figures 1 and 2). All tumor markers were within normal range.

Core needle aspiration was not performed because the lesion was so consistent with liposarcoma in MRI. The patient was a young woman with firm willing for preserving the fetus. After thorough consultation with radiologist, obstetrician, urologist, and anesthesiologist, considering the MRI revealed no clear evidence of major organ and vessel involvement, and the patient was in her second trimester, we decided to perform the en bloc resection with an attempt for fetus preservation.

**Figure 3:**
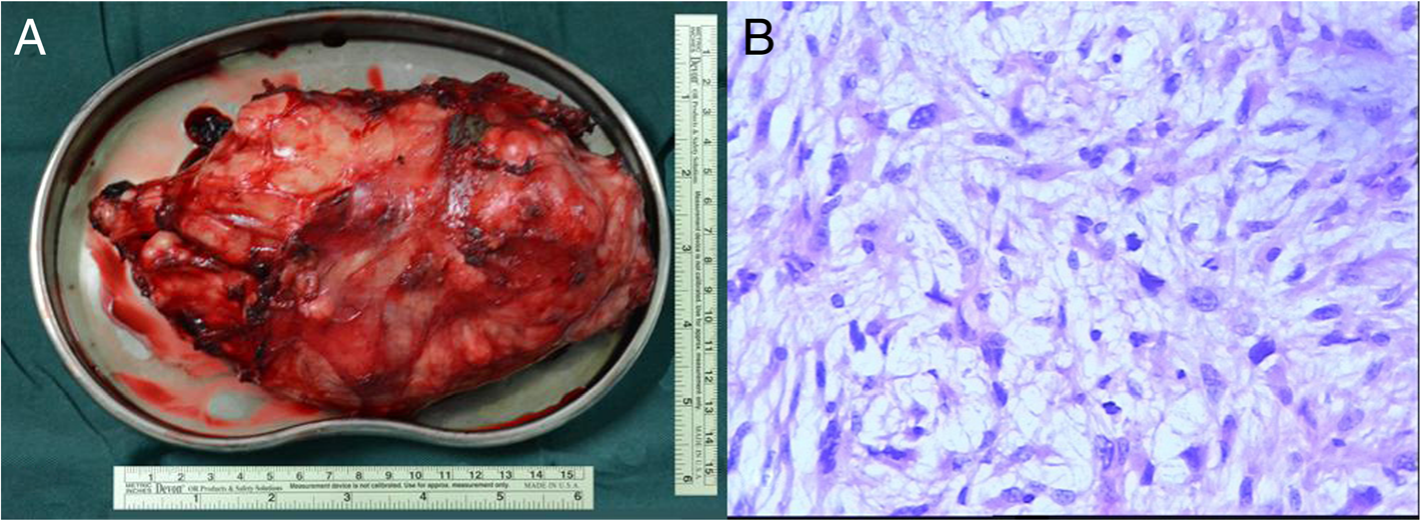
**Macroscopic view and histologic section.** The macroscopic view of the en bloc resected specimen **(3A)**. The histologic section shows fusiform cell subtype of myxoid liposarcoma (magnification of × 200) **(3B)**.

**Figure 4:**
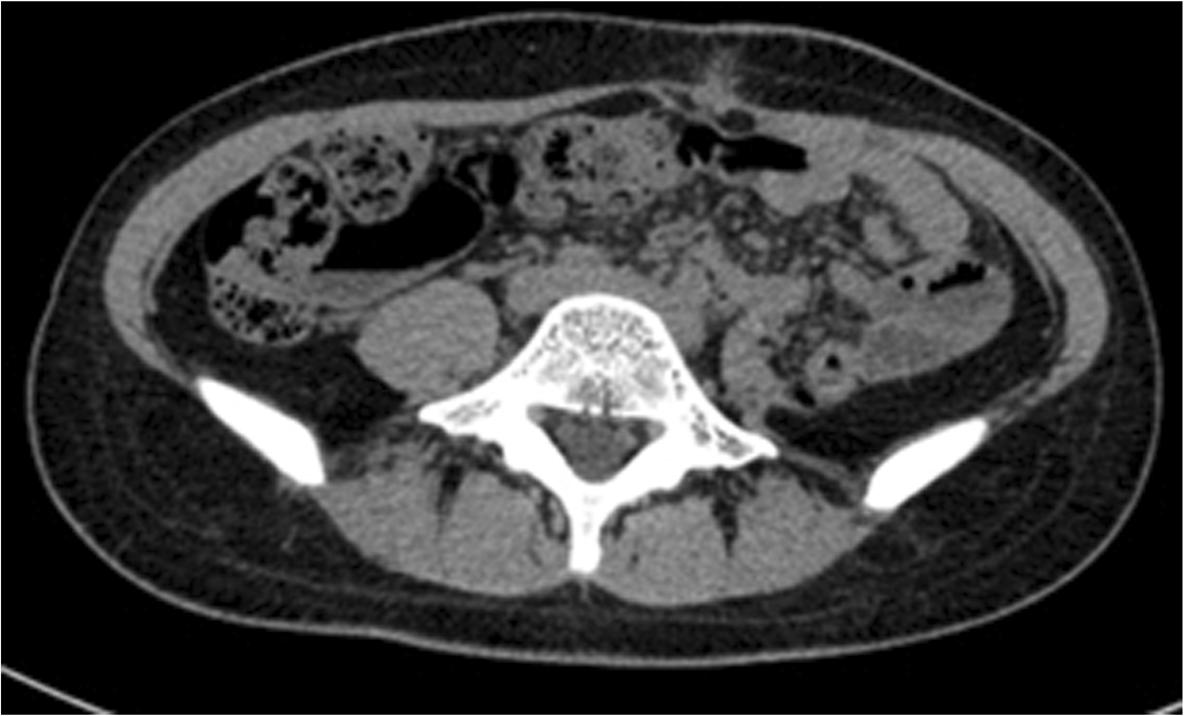
**Follow-up CT 6 months following the surgery.**

The laparotomy was performed with left paramedian incision under general anesthesia at 20 weeks of gestation. The giant retroperitoneal tumor located in the left retroperitoneal space extended from the hilum of the left kidney to the left pelvis, without involvement of adjacent muscle, kidney, intestine, and major vessels. The en bloc resection was accomplished with negative surgical margin. The operation lasts 3 h, and intraoperative hemorrhage was 450 ml, with transfusion of 2 u of packed red blood cell. Fetal heart rate under perioperative ultrasonography monitoring ranged from 130 to 150 bpm. Retodrine was administered until 1 week postoperatively. The patient had an uneventful recovery. The final pathology demonstrated a low-grade myxoid liposarcoma with negative surgical margin, mitotic index 5/50 HPF. IHC results showed the following: desmin (−), Ki-67 (<15%), NF (−), vimentin (+), S-100 (−), SMA (−), MyoD1 (−), CD34 (−), CD117 (−), NeuN (−), P16 (−), HMB45 (−), CD68 (−), CD31 (−), calretinin (−), calponin (−) (Figure 3). The patient had a planned cesarean section at 37 weeks of gestation and delivered a healthy baby. The patient was under regular follow-up, a CT was taken 6 months following the surgery, and no recurrence was detected (Figure 4).

## Discussion

RPLS is one of the most common types of soft tissue sarcoma with peak prevalence between the age of 40 to 70 years, which is usually seen in male 1.43:1 [1,2]. RPLS during pregnancy is extremely rare and a challenge for both the surgeon and pregnant woman [3].

Liposarcoma is categorized into five histological subtypes: well-differentiated, myxoid, round-cell, pleomorphic, and dedifferentiated (WHO classification, 2002). Well-differentiated and myxoid subtypes belong to the low-grade lesions, whereas the other subtypes are considered as high-grade lesions. The prognosis of liposarcoma is determined by its histologic subtype, resection margin, contiguous organ resection, and age of the patients [2].

Myxoid liposarcoma is the second most common subtype, consists of 18% of all liposarcoma, and is the most common subtype in young patients [4]. It is often found with painless palpable mass arising in the thigh and retroperitoneal space, especially for young patients [4]. MRI is a highly reliable radiological method in diagnosing these tumors, especially for assessment of pregnant woman. With careful interpretation, the MRI can provide us with enough information for accurate diagnosis differentiation and even identification of subtypes [5-7]. Myxoid liposarcoma has a relatively characteristic appearance as a well-circumscribed multinodular mass with low signal intensity on T1WI and a high but heterogeneous intensity on T2WI.

The relationship between pregnancy and liposarcoma is not clear. Cantin and McNeer considered that pregnancy does not adversely affect the prognosis of the tumor. Whereas, they suggested that an estrogen-progesterone environment possibly has a favorable impact on the natural history of sarcoma, and hormone therapy in the management of metastatic sarcoma should be explored [8].

The retroperitoneal space is rather vast; RPLS has no specific symptoms in the early stage. Although surgery is the mainstay of the therapeutic modalities, the RPLS is usually diagnosed in the advanced stage with a palpable mass, and contiguous organ resection is often indicated [9]. If resected with negative surgical margin, the 5-year overall survival rate can be 68% to 80%, although local recurrence rate can be as high as 75% [2,9]. The extended surgery, contiguous organ resection, major vessel resection, and reconstruction are often indicated to get a negative surgical margin and a better prognosis [10].

This patient was diagnosed with giant retroperitoneal tumor incidentally during her routine obstetric examination. The therapeutic protocol is challenging because: 1. The patient has a firm willing of preserving the fetus; 2. The en bloc resection is urgent as the tumor is close to the hilum of left kidney, and waiting for maturation of the fetus may result in contiguous kidney resection; 3. The impact on the fetus should be assessed such as time of gestation, hemorrhage, length of operation, medications, and anesthesia.

We reviewed the literature; only 16 cases of liposarcoma during pregnancy were reported in English literature, ten were derived from retroperitoneal space, and five cases are myxoid subtype. Surgery was performed in five cases after delivery, five cases concurrent with cesarean section. Three patients died within the first year after surgery [11-22]. In only two cases, surgeries were performed during pregnancy in 13 weeks of gestation [17,19]. Most surgery were performed postpartum or at the time of cesarean section.

## Conclusions

We presented a rare case of giant RPLS during pregnancy. The surgeon should consider the patient’s willingness, times of gestation, extension of surgery, multidisciplinary collaboration, and finally making of an individualized surgical plan. En bloc surgery with preservation of the fetus in the late second trimester could be feasible. As the tumor has a high risk of local recurrence, lifetime follow-up is indicated.

## Consent

Written informed consent was obtained from the patient for publication of this case report and accompanying images. A copy of the written consent is available for review by the Editor-in-Chief of this journal. The treatment was approved by the medical ethics committees of the PLA General Hospital.

## Abbreviation

RPLS: retroperitoneal liposarcoma
MRI: magnetic resonance imaging
